# Supplementary material for: Impact of implementation of the national institute for health and clinical excellence (NICE) head injury guideline in a tertiary care center emergency department: A pre and post-intervention study
Source: PLoS One. 2021 Jul 15;16(7):e0254754. doi: 10.1371/journal.pone.0254754 (PMC8282013; doi:10.1371/journal.pone.0254754)
Supplement: S1 File — (PDF) [file pone.0254754.s001.pdf]

Head Injury in Dhulikhel Emergency Department.

Please give your sincere opinion regarding the cases listed below, regarding whether to indicate a head CT or not for the given incident, symptoms and signs. For each case, also give your confidence level regarding your recommendation of indicating head CT

* Required

CASE - 1

Age:	11 months
Triage zone:	Red
Mode of injury	RTA
Brief History	Laceration 6 * 2 cm in head
Time of Incidence	7:00 AM
Time of Presentation	8:00 AM
Airway	Patent, SpO2 - 98% in room air
Breathing	Bilateral equal air entry, RR - 30/m
Circulation	PR - 88/m, BP - 120/60 mm HG, CRT <2 seconds
GCS at presentation	14
GCS at 2 hours of injury	14
Pupils -	Bilateral equal and reactive.
Compression	Chest – Negative<br>Pelvis - Negative
Exposure -	No spine tenderness.
EFAST -	Negative
Any sign of basal skull fracture	+
Post- traumatic seizure	-
Vomiting	1
Loss of consciousness (witnessed)	-
Abnormal drowsiness	-
H/o bleeding or clotting disorders	-
Amnesia	-

1. Is head CT required in this case?

Mark only one oval.

- ⬭ Yes *Skip to question 2*
- ⬭ No *Skip to question 4*

If you are demanding a CT head for this patient,

2. What is the indication according to your opinion? *

3. How confident are you to demand head CT in this case? *

Mark only one oval.

	1	2	3	4	5	6	7	8	9	10	
Low	◯	◯	◯	◯	◯	◯	◯	◯	◯	◯	High

Skip to question 5

If you are not demanding head CT in this case,

4. How confident do you feel to not demand head CT in this case? *

Mark only one oval.

	1	2	3	4	5	6	7	8	9	10	
Low	◯	◯	◯	◯	◯	◯	◯	◯	◯	◯	High

CASE - 2

Age	25 years
Triage zone	Red
Mode of injury	Physical assault
Brief History	Laceration 8 * 1 cm in head
Time of Incidence	6:30 AM
Time of Presentation	7:00 AM
Airway	Patent, SpO2 - 98% in room air
Breathing	Bilateral equal air entry, RR - 22/m
Circulation	PR - 80/m, BP - 130/70 mm HG, CRT <2 seconds
GCS at presentation	15
GCS at 2 hours of injury	15
Pupils -	Bilateral equal and reactive.
Compression -	Chest – Negative
	Pelvis – Negative
Exposure -	No spine tenderness.
EFAST -	Negative
Any sign of basal skull fracture	-
Post- traumatic seizure	-
Vomiting	2 episodes
Loss of consciousness (witnessed)	-
Abnormal drowsiness	-
H/o bleeding or clotting disorders	-
Amnesia	-

5. Is head CT required in this case? *

Mark only one oval.

- ( ) Yes *Skip to question 6*
- ( ) No *Skip to question 8*

If you are demanding a CT head for this patient,

6. What is the indication according to your opinion? *

____

7. How confident are you to demand head CT in this case? *

Mark only one oval.

	1	2	3	4	5	6	7	8	9	10	
Low	◯	◯	◯	◯	◯	◯	◯	◯	◯	◯	High

Skip to question 9

If you are not demanding head CT in this case,

8. How confident do you feel to not demand head CT in this case? *

Mark only one oval.

	1	2	3	4	5	6	7	8	9	10	
Low	◯	◯	◯	◯	◯	◯	◯	◯	◯	◯	High

CASE - 3

Age	16 years
Triage zone	Yellow
Mode of injury	RTA
Brief History	Ejected from passenger seat of bike
Time of Incidence	12:30 PM
Time of Presentation	3:30 PM
Airway	Patent, SpO2 - 98% in room air
Breathing	Bilateral equal air entry, RR - 24/m
Circulation	PR - 78/m, BP - 120/80 mm HG, CRT <2 seconds
GCS at presentation	15
GCS at 2 hours of injury	15
Pupils -	Bilateral equal and reactive.
Compression -	Chest – Negative
	Pelvis – Negative
Exposure -	No spine tenderness.
EFAST -	Negative
Any sign of basal skull fracture	-
Post- traumatic seizure	-
Vomiting	-
Loss of consciousness (witnessed)	15 minutes
Abnormal drowsiness	+
H/o bleeding or clotting disorders	-
Amnesia	-

9. Is head CT required in this case? *

Mark only one oval.

- ◯ Yes *Skip to question 10*
- ◯ No *Skip to question 12*

If you are demanding a CT head for this patient,

10. What is the indication according to your opinion? *

____

11. How confident are you to demand head CT in this case? *

Mark only one oval.

	1	2	3	4	5	6	7	8	9	10	
Low											High

Skip to question 13

If you are not demanding head CT in this case,

12. How confident do you feel to not demand head CT in this case? *

Mark only one oval.

	1	2	3	4	5	6	7	8	9	10	
Low											High

CASE - 4

Age	42 years
Triage zone	Yellow
Mode of injury	RTA
Brief History	Stuck in passenger seat of car
Time of Incidence	2:30 PM
Time of Presentation	3:00 PM
Airway	Patent, SpO2 - 94% in room air
Breathing	Bilateral equal air entry, RR - 22/m
Circulation	PR - 80/m, BP - 130/70 mm HG, CRT <2 seconds
GCS at presentation	15
GCS at 2 hours of injury	15
Pupils -	Bilateral equal and reactive.
Compression -	Chest – Negative<br>Pelvis – Negative
Exposure -	No spine tenderness.
EFAST -	Negative
Any sign of basal skull fracture	-
Post- traumatic seizure	-
Vomiting	-
Loss of consciousness (witnessed)	5 minutes
Abnormal drowsiness	-
H/o bleeding or clotting disorders	-
Amnesia	-

13. Is head CT required in this case? *

Mark only one oval.

- ( ) Yes *Skip to question 14*
- ( ) No *Skip to question 16*

If you are demanding a CT head for this patient,

14. What is the indication according to your opinion? *

____

15. How confident are you to demand head CT in this case? *

Mark only one oval.

	1	2	3	4	5	6	7	8	9	10	
Low	⬭	⬭	⬭	⬭	⬭	⬭	⬭	⬭	⬭	⬭	High

Skip to question 17

If you are not demanding head CT in this case,

16. How confident do you feel to not demand head CT in this case? *

Mark only one oval.

	1	2	3	4	5	6	7	8	9	10	
Low	⬭	⬭	⬭	⬭	⬭	⬭	⬭	⬭	⬭	⬭	High

CASE - 5

Age	13 years
Triage zone	Orange
Mode of injury	Fall (4 metres)
Brief History	Abrasion over frontal region
Time of Incidence	3:30 PM
Time of Presentation	4:00 PM
Airway	Patent, SpO2 - 90% in room air
Breathing	Bilateral equal air entry, RR - 16/m
Circulation	PR - 84/m, BP - 110/60 mm HG, CRT <2 seconds
GCS at presentation	13
GCS at 2 hours of injury	13
Pupils -	Bilateral equal and reactive.
Compression -	Chest – Negative
	Pelvis – Negative
Exposure -	No spine tenderness.
EFAST -	Negative
Any sign of basal skull fracture	-
Post- traumatic seizure	-
Vomiting	-
Loss of consciousness (witnessed)	-
Abnormal drowsiness	-
H/o bleeding or clotting disorders	-
Amnesia	-

17. Is head CT required in this case? *

Mark only one oval.

- Yes *Skip to question 18*
- No *Skip to question 20*

If you are demanding a CT head for this patient,

18. What is the indication according to your opinion? *

____

19. How confident are you to demand head CT in this case? *

Mark only one oval.

	1	2	3	4	5	6	7	8	9	10	
Low	( )	( )	( )	( )	( )	( )	( )	( )	( )	( )	High

Skip to question 21

If you are not demanding head CT in this case,

20. How confident do you feel to not demand head CT in this case? *

Mark only one oval.

	1	2	3	4	5	6	7	8	9	10	
Low	( )	( )	( )	( )	( )	( )	( )	( )	( )	( )	High

CASE - 6

Age	55 years
Triage zone	Yellow
Mode of injury	Fall (level ground)
Brief History	Injury over left temporal region. No external wounds
Time of Incidence	5:00 PM
Time of Presentation	9:00 PM
Airway	Patent, SpO2 - 98% in room air
Breathing	Bilateral equal air entry, RR - 22/m
Circulation	PR - 70/m, BP - 130/70 mm HG, CRT <2 seconds
GCS at presentation	12
GCS at 2 hours of injury	12
Pupils -	Bilateral equal and reactive.
Compression -	Chest – Negative
	Pelvis – Negative
Exposure -	No spine tenderness.
EFAST -	Negative
Any sign of basal skull fracture	-
Post- traumatic seizure	-
Vomiting	-
Loss of consciousness (witnessed)	-
Abnormal drowsiness	-
H/o bleeding or clotting disorders	-
Amnesia	-

21. Is head CT required in this case? *

Mark only one oval.

- Yes *Skip to question 22*
- No *Skip to question 24*

If you are demanding a CT head for this patient,

22. What is the indication according to your opinion? *

____

23. How confident are you to demand head CT in this case? *

Mark only one oval.

	1	2	3	4	5	6	7	8	9	10	
Low	⬭	⬭	⬭	⬭	⬭	⬭	⬭	⬭	⬭	⬭	High

Skip to question 25

If you are not demanding head CT in this case,

24. How confident do you feel to not demand head CT in this case? *

Mark only one oval.

	1	2	3	4	5	6	7	8	9	10	
Low	⬭	⬭	⬭	⬭	⬭	⬭	⬭	⬭	⬭	⬭	High

CASE - 7

Age	70 years
Triage zone	Orange
Mode of injury	Fall injury - 7 stairs
Brief History	Swelling over left parietal region.

Time of Incidence	4:00 AM
Time of Presentation	4:45 AM

Airway	Patent, SpO2 - 92% in room air
Breathing	Bilateral equal air entry, RR - 24/m
Circulation	PR - 82/m, BP - 150/80 mm HG, CRT <2 seconds

GCS at presentation	15
GCS at 2 hours of injury	15

Pupils -	Bilateral equal and reactive.
Compression -	Chest – Negative
	Pelvis – Negative
Exposure -	No spine tenderness.
EFAST -	Negative

Any sign of basal skull fracture	-
Post- traumatic seizure	-
Vomiting	-
Loss of consciousness (witnessed)	-
Abnormal drowsiness	-
H/o bleeding or clotting disorders	-
Amnesia	-

25. Is head CT required in this case? *

Mark only one oval.

- ( ) Yes *Skip to question 26*
- ( ) No *Skip to question 28*

If you are demanding a CT head for this patient,

26. What is the indication according to your opinion? *

____

27. How confident are you to demand head CT in this case? *

Mark only one oval.

	1	2	3	4	5	6	7	8	9	10	
Low	◯	◯	◯	◯	◯	◯	◯	◯	◯	◯	High

Skip to question 29

If you are not demanding head CT in this case,

28. How confident do you feel to not demand head CT in this case? *

Mark only one oval.

	1	2	3	4	5	6	7	8	9	10	
Low	◯	◯	◯	◯	◯	◯	◯	◯	◯	◯	High

CASE - 8

Age	2 years
Triage zone	Red
Mode of injury	Fall (2 metres)
Brief History	Injury over frontal region, swelling and crepitation present
Time of Incidence	2:30 PM
Time of Presentation	5:00 PM
Airway	Patent, SpO2 - 98% in room air
Breathing	Bilateral equal air entry, RR - 22/m
Circulation	PR - 78/m, BP - 130/70 mm HG, CRT <2 seconds
GCS at presentation	15
GCS at 2 hours of injury	15
Pupils -	Bilateral equal and reactive.
Compression -	Chest – Negative<br>Pelvis – Negative
Exposure -	No spine tenderness.
EFAST -	Negative
Any sign of basal skull fracture	-
Post- traumatic seizure	-
Vomiting	4
Loss of consciousness (witnessed)	-
Abnormal drowsiness	-
H/o bleeding or clotting disorders	-
Amnesia	-

29. Is head CT required in this case? *

Mark only one oval.

- ( ) Yes *Skip to question 30*
- ( ) No *Skip to question 32*

If you are demanding a CT head for this patient,

30. What is the indication according to your opinion? *

____

31. How confident are you to demand head CT in this case? *

Mark only one oval.

	1	2	3	4	5	6	7	8	9	10	
Low	◯	◯	◯	◯	◯	◯	◯	◯	◯	◯	High

Skip to question 33

If you are not demanding head CT in this case,

32. How confident do you feel to not demand head CT in this case? *

Mark only one oval.

	1	2	3	4	5	6	7	8	9	10	
Low	◯	◯	◯	◯	◯	◯	◯	◯	◯	◯	High

CASE - 9

Age	50 years
Triage zone	Red
Mode of injury	Fall (7 metres)
Brief History	Abrasion over frontal region

Time of Incidence	4:00 PM
Time of Presentation	7:00 PM

Airway	Patent, SpO2 - 95% in room air
Breathing	Bilateral equal air entry, RR - 16/m
Circulation	PR - 80/m, BP - 110/60 mm HG, CRT <2 seconds

GCS at presentation	15
GCS at 2 hours of injury	15

Pupils -	Bilateral equal and reactive.
Compression -	Chest – Negative
	Pelvis – Negative
Exposure -	No spine tenderness.
EFAST -	Negative

Any sign of basal skull fracture	-
Post- traumatic seizure	1 episode
Vomiting	-
Loss of consciousness (witnessed)	-
Abnormal drowsiness	-
H/o bleeding or clotting disorders	-
Amnesia	-

33. Is head CT required in this case? *

Mark only one oval.

- ( ) Yes *Skip to question 34*
- ( ) No *Skip to question 36*

If you are demanding a CT head for this patient,

34. What is the indication according to your opinion? *

____

35. How confident are you to demand head CT in this case? *

Mark only one oval.

	1	2	3	4	5	6	7	8	9	10	
Low	◯	◯	◯	◯	◯	◯	◯	◯	◯	◯	High

Skip to question 37

If you are not demanding head CT in this case,

36. How confident do you feel to not demand head CT in this case? *

Mark only one oval.

	1	2	3	4	5	6	7	8	9	10	
Low	◯	◯	◯	◯	◯	◯	◯	◯	◯	◯	High

CASE - 10

Age	37 years
Triage zone	Orange
Mode of injury	Hit by rock
Brief History	Injury over occipital region, swelling and crepitation present
Time of Incidence	8:30 PM
Time of Presentation	11:00 PM
Airway	Patent, SpO2 - 90% in room air
Breathing	Bilateral equal air entry, RR - 16/m
Circulation	PR - 88/m, BP - 120/60 mm HG, CRT <2 seconds
GCS at presentation	11
GCS at 2 hours of injury	11
Pupils -	Bilateral equal and reactive.
Compression -	Chest – Negative<br>Pelvis – Negative
Exposure -	No spine tenderness.
EFAST -	Negative
Any sign of basal skull fracture	-
Post- traumatic seizure	-
Vomiting	-
Loss of consciousness (witnessed)	-
Abnormal drowsiness	-
H/o bleeding or clotting disorders	-
Amnesia	-

37. Is head CT required in this case? *

Mark only one oval.

- Yes *Skip to question 38*
- No *Skip to question 40*

If you are demanding a CT head for this patient,

38. What is the indication according to your opinion? *

____

39. How confident are you to demand head CT in this case? *

Mark only one oval.

	1	2	3	4	5	6	7	8	9	10	
Low	⬭	⬭	⬭	⬭	⬭	⬭	⬭	⬭	⬭	⬭	High

Skip to question 41

If you are not demanding head CT in this case,

40. How confident do you feel to not demand head CT in this case? *

Mark only one oval.

	1	2	3	4	5	6	7	8	9	10	
Low	⬭	⬭	⬭	⬭	⬭	⬭	⬭	⬭	⬭	⬭	High

CASE - 11

Age	2 months
Triage zone	Red
Mode of injury	Fall (level ground)
Brief History	Injury over left temporal region. No external wounds

Time of Incidence 8:00 PM
Time of Presentation 8:40 PM

Airway	Patent, SpO2 - 98% in room air
Breathing	Bilateral equal air entry, RR - 32/m
Circulation	PR - 78/m, BP - 100/70 mm HG, CRT <2 seconds

GCS at presentation 15
GCS at 2 hours of injury 13

Pupils -	Bilateral equal and reactive.
Compression -	Chest – Negative
	Pelvis – Negative
Exposure -	No spine tenderness.
EFAST -	Negative

Any sign of basal skull fracture	-
Post- traumatic seizure	2 episodes
Vomiting	-
Loss of consciousness (witnessed)	-
Abnormal drowsiness	-
H/o bleeding or clotting disorders	-
Amnesia	-

41. Is head CT required in this case? *

Mark only one oval.

- Yes *Skip to question 42*
- No *Skip to question 44*

If you are demanding a CT head for this patient,

42. What is the indication according to your opinion? *

____

43. How confident are you to demand head CT in this case? *

Mark only one oval.

	1	2	3	4	5	6	7	8	9	10	
Low	⬭	⬭	⬭	⬭	⬭	⬭	⬭	⬭	⬭	⬭	High

Skip to question 45

If you are not demanding head CT in this case,

44. How confident do you feel to not demand head CT in this case? *

Mark only one oval.

	1	2	3	4	5	6	7	8	9	10	
Low	⬭	⬭	⬭	⬭	⬭	⬭	⬭	⬭	⬭	⬭	High

CASE - 12

Age	65 years
Triage zone	Red
Mode of injury	Physical assault
Brief History	Multiple bruises over head and body

Time of Incidence 12:00 PM
Time of Presentation 1:00 PM

Airway	Patent, SpO2 - 90% in room air
Breathing	Bilateral equal air entry, RR - 16/m
Circulation	PR - 70/m, BP - 110/60 mm HG, CRT <2 seconds

GCS at presentation 14
GCS at 2 hours of injury 14

Pupils -	Bilateral equal and reactive.
Compression -	Chest – Negative
	Pelvis – Negative
Exposure -	No spine tenderness.
EFAST -	Negative

Any sign of basal skull fracture	-
Post- traumatic seizure	-
Vomiting	-
Loss of consciousness (witnessed)	-
Abnormal drowsiness	-
H/o bleeding or clotting disorders	+
Amnesia	1 hour

45. Is head CT required in this case? *

Mark only one oval.

- ◯ Yes *Skip to question 46*
- ◯ No *Skip to question 48*

If you are demanding a CT head for this patient,

46. What is the indication according to your opinion? *

____

47. How confident are you to demand head CT in this case? *

Mark only one oval.

	1	2	3	4	5	6	7	8	9	10	
Low	◯	◯	◯	◯	◯	◯	◯	◯	◯	◯	High

If you are not demanding head CT in this case,

48. How confident do you feel to not demand head CT in this case? *

Mark only one oval.

	1	2	3	4	5	6	7	8	9	10	
Low	◯	◯	◯	◯	◯	◯	◯	◯	◯	◯	High

This content is neither created nor endorsed by Google.

Google Forms
