# Supplementary material for: Impact of implementation of the national institute for health and clinical excellence (NICE) head injury guideline in a tertiary care center emergency department: A pre and post-intervention study
Source: PLoS One. 2021 Jul 15;16(7):e0254754. doi: 10.1371/journal.pone.0254754 (PMC8282013; doi:10.1371/journal.pone.0254754)
Supplement: S2 File — (PDF) [file pone.0254754.s002.pdf]

Head Injury in Dhulikhel Emergency Department.

Please give your sincere opinion regarding the cases listed below, regarding whether to indicate a head CT or not for the given incident, symptoms and signs. For each case, also give your confidence level regarding your recommendation of indicating head CT

* Required

1. Email address *

CASE - 1

Age: 11 months
Triage zone: Red
Mode of injury RTA
Brief History Laceration 6 * 2 cm in head

Time of Incidence 7:00 AM
Time of Presentation 8:00 AM

Airway Patent, SpO2 - 98% in room air
Breathing Bilateral equal air entry, RR - 30/m
Circulation PR - 88/m, BP - 120/60 mm HG, CRT <2 seconds

GCS at presentation 14
GCS at 2 hours of injury 14

Pupils - Bilateral equal and reactive.
Compression Chest – Negative
Pelvis - Negative
Exposure - No spine tenderness.
EFAST - Negative

Any sign of basal skull fracture +
Post- traumatic seizure -
Vomiting 1
Loss of consciousness (witnessed) -
Abnormal drowsiness -
H/o bleeding or clotting disorders -
Amnesia -

2. Is head CT required in this case?

Mark only one oval.

- ◯ Yes *Skip to question 3*
- ◯ No *Skip to question 5*

If you are demanding a CT head for this patient,

3. What is the indication according to your opinion? *

____

4. How confident are you to demand head CT in this case? *

Mark only one oval.

	1	2	3	4	5	6	7	8	9	10	
Low	◯	◯	◯	◯	◯	◯	◯	◯	◯	◯	High

Skip to question 6

If you are not demanding head CT in this case,

5. How confident do you feel to not demand head CT in this case? *

Mark only one oval.

	1	2	3	4	5	6	7	8	9	10	
Low	◯	◯	◯	◯	◯	◯	◯	◯	◯	◯	High

CASE - 2

Age	25 years
Triage zone	Red
Mode of injury	Physical assault
Brief History	Laceration 8 * 1 cm in head

Time of Incidence	6:30 AM
Time of Presentation	7:00 AM

Airway	Patent, SpO2 - 98% in room air
Breathing	Bilateral equal air entry, RR - 22/m
Circulation	PR - 80/m, BP - 130/70 mm HG, CRT <2 seconds

GCS at presentation	15
GCS at 2 hours of injury	15

Pupils -	Bilateral equal and reactive.
Compression -	Chest – Negative
	Pelvis – Negative
Exposure -	No spine tenderness.
EFAST -	Negative

Any sign of basal skull fracture	-
Post- traumatic seizure	-
Vomiting	2 episodes
Loss of consciousness (witnessed)	-
Abnormal drowsiness	-
H/o bleeding or clotting disorders	-
Amnesia	-

6. Is head CT required in this case? *

Mark only one oval.

- Yes *Skip to question 7*
- No *Skip to question 9*

If you are demanding a CT head for this patient,

7. What is the indication according to your opinion? *

____

8. How confident are you to demand head CT in this case? *

Mark only one oval.

	1	2	3	4	5	6	7	8	9	10	
Low	◯	◯	◯	◯	◯	◯	◯	◯	◯	◯	High

Skip to question 10

If you are not demanding head CT in this case,

9. How confident do you feel to not demand head CT in this case? *

Mark only one oval.

	1	2	3	4	5	6	7	8	9	10	
Low	◯	◯	◯	◯	◯	◯	◯	◯	◯	◯	High

CASE - 3

Age	16 years
Triage zone	Yellow
Mode of injury	RTA
Brief History	Ejected from passenger seat of bike
Time of Incidence	12:30 PM
Time of Presentation	3:30 PM
Airway	Patent, SpO2 - 98% in room air
Breathing	Bilateral equal air entry, RR - 24/m
Circulation	PR - 78/m, BP - 120/80 mm HG, CRT <2 seconds
GCS at presentation	15
GCS at 2 hours of injury	15
Pupils -	Bilateral equal and reactive.
Compression -	Chest – Negative
	Pelvis – Negative
Exposure -	No spine tenderness.
EFAST -	Negative
Any sign of basal skull fracture	-
Post- traumatic seizure	-
Vomiting	-
Loss of consciousness (witnessed)	15 minutes
Abnormal drowsiness	+
H/o bleeding or clotting disorders	-
Amnesia	-

10. Is head CT required in this case? *

Mark only one oval.

- ⬭ Yes *Skip to question 11*
- ⬭ No *Skip to question 13*

If you are demanding a CT head for this patient,

11. What is the indication according to your opinion? *

____

12. How confident are you to demand head CT in this case? *

Mark only one oval.

	1	2	3	4	5	6	7	8	9	10	
Low	◯	◯	◯	◯	◯	◯	◯	◯	◯	◯	High

Skip to question 14

If you are not demanding head CT in this case,

13. How confident do you feel to not demand head CT in this case? *

Mark only one oval.

	1	2	3	4	5	6	7	8	9	10	
Low	◯	◯	◯	◯	◯	◯	◯	◯	◯	◯	High

CASE - 4

Age	42 years
Triage zone	Yellow
Mode of injury	RTA
Brief History	Stuck in passenger seat of car
Time of Incidence	2:30 PM
Time of Presentation	3:00 PM
Airway	Patent, SpO2 - 94% in room air
Breathing	Bilateral equal air entry, RR - 22/m
Circulation	PR - 80/m, BP - 130/70 mm HG, CRT <2 seconds
GCS at presentation	15
GCS at 2 hours of injury	15
Pupils -	Bilateral equal and reactive.
Compression -	Chest – Negative<br>Pelvis – Negative
Exposure -	No spine tenderness.
EFAST -	Negative
Any sign of basal skull fracture	-
Post- traumatic seizure	-
Vomiting	-
Loss of consciousness (witnessed)	5 minutes
Abnormal drowsiness	-
H/o bleeding or clotting disorders	-
Amnesia	-

14. Is head CT required in this case? *

Mark only one oval.

- ( ) Yes *Skip to question 15*
- ( ) No *Skip to question 17*

If you are demanding a CT head for this patient,

15. What is the indication according to your opinion? *

____

16. How confident are you to demand head CT in this case? *

Mark only one oval.

	1	2	3	4	5	6	7	8	9	10	
Low	⬭	⬭	⬭	⬭	⬭	⬭	⬭	⬭	⬭	⬭	High

Skip to question 18

If you are not demanding head CT in this case,

17. How confident do you feel to not demand head CT in this case? *

Mark only one oval.

	1	2	3	4	5	6	7	8	9	10	
Low	⬭	⬭	⬭	⬭	⬭	⬭	⬭	⬭	⬭	⬭	High

CASE - 5

Age	13 years
Triage zone	Orange
Mode of injury	Fall (4 metres)
Brief History	Abrasion over frontal region
Time of Incidence	3:30 PM
Time of Presentation	4:00 PM
Airway	Patent, SpO2 - 90% in room air
Breathing	Bilateral equal air entry, RR - 16/m
Circulation	PR - 84/m, BP - 110/60 mm HG, CRT <2 seconds
GCS at presentation	13
GCS at 2 hours of injury	13
Pupils -	Bilateral equal and reactive.
Compression -	Chest – Negative<br>Pelvis – Negative
Exposure -	No spine tenderness.
EFAST -	Negative
Any sign of basal skull fracture	-
Post- traumatic seizure	-
Vomiting	-
Loss of consciousness (witnessed)	-
Abnormal drowsiness	-
H/o bleeding or clotting disorders	-
Amnesia	-

18. Is head CT required in this case? *

Mark only one oval.

- Yes *Skip to question 19*
- No *Skip to question 21*

If you are demanding a CT head for this patient,

19. What is the indication according to your opinion? *

____

20. How confident are you to demand head CT in this case? *

Mark only one oval.

	1	2	3	4	5	6	7	8	9	10	
Low											High

Skip to question 22

If you are not demanding head CT in this case,

21. How confident do you feel to not demand head CT in this case? *

Mark only one oval.

	1	2	3	4	5	6	7	8	9	10	
Low											High

CASE - 6

Age 55 years
Triage zone Yellow
Mode of injury Fall (level ground)
Brief History Injury over left temporal region. No external wounds

Time of Incidence 5:00 PM
Time of Presentation 9:00 PM

Airway Patent, SpO2 - 98% in room air
Breathing Bilateral equal air entry, RR - 22/m
Circulation PR - 70/m, BP - 130/70 mm HG, CRT <2 seconds

GCS at presentation 12
GCS at 2 hours of injury 12

Pupils - Bilateral equal and reactive.
Compression - Chest – Negative
Pelvis – Negative
Exposure - No spine tenderness.
EFAST - Negative

Any sign of basal skull fracture -
Post- traumatic seizure -
Vomiting -
Loss of consciousness (witnessed) -
Abnormal drowsiness -
H/o bleeding or clotting disorders -
Amnesia -

22. Is head CT required in this case? *

Mark only one oval.

- Yes *Skip to question 23*
- No *Skip to question 25*

If you are demanding a CT head for this patient,

23. What is the indication according to your opinion? *

____

24. How confident are you to demand head CT in this case? *

Mark only one oval.

	1	2	3	4	5	6	7	8	9	10	
Low	⬭	⬭	⬭	⬭	⬭	⬭	⬭	⬭	⬭	⬭	High

Skip to question 26

If you are not demanding head CT in this case,

25. How confident do you feel to not demand head CT in this case? *

Mark only one oval.

	1	2	3	4	5	6	7	8	9	10	
Low	⬭	⬭	⬭	⬭	⬭	⬭	⬭	⬭	⬭	⬭	High

CASE - 7

Age	70 years
Triage zone	Orange
Mode of injury	Fall injury - 7 stairs
Brief History	Swelling over left parietal region.

Time of Incidence 4:00 AM
Time of Presentation 4:45 AM

Airway	Patent, SpO2 - 92% in room air
Breathing	Bilateral equal air entry, RR - 24/m
Circulation	PR - 82/m, BP - 150/80 mm HG, CRT <2 seconds

GCS at presentation 15
GCS at 2 hours of injury 15

Pupils -	Bilateral equal and reactive.
Compression -	Chest – Negative
	Pelvis – Negative
Exposure -	No spine tenderness.
EFAST -	Negative

Any sign of basal skull fracture	-
Post- traumatic seizure	-
Vomiting	-
Loss of consciousness (witnessed)	-
Abnormal drowsiness	-
H/o bleeding or clotting disorders	-
Amnesia	-

26. Is head CT required in this case? *

Mark only one oval.

- ( ) Yes *Skip to question 27*
- ( ) No *Skip to question 29*

If you are demanding a CT head for this patient,

27. What is the indication according to your opinion? *

____

28. How confident are you to demand head CT in this case? *

Mark only one oval.

	1	2	3	4	5	6	7	8	9	10	
Low	◯	◯	◯	◯	◯	◯	◯	◯	◯	◯	High

Skip to question 30

If you are not demanding head CT in this case,

29. How confident do you feel to not demand head CT in this case? *

Mark only one oval.

	1	2	3	4	5	6	7	8	9	10	
Low	◯	◯	◯	◯	◯	◯	◯	◯	◯	◯	High

CASE - 8

Age	2 years
Triage zone	Red
Mode of injury	Fall (2 metres)
Brief History	Injury over frontal region, swelling and crepitation present
Time of Incidence	2:30 PM
Time of Presentation	5:00 PM
Airway	Patent, SpO2 - 98% in room air
Breathing	Bilateral equal air entry, RR - 22/m
Circulation	PR - 78/m, BP - 130/70 mm HG, CRT <2 seconds
GCS at presentation	15
GCS at 2 hours of injury	15
Pupils -	Bilateral equal and reactive.
Compression -	Chest – Negative
	Pelvis – Negative
Exposure -	No spine tenderness.
EFAST -	Negative
Any sign of basal skull fracture	-
Post- traumatic seizure	-
Vomiting	4
Loss of consciousness (witnessed)	-
Abnormal drowsiness	-
H/o bleeding or clotting disorders	-
Amnesia	-

30. Is head CT required in this case? *

Mark only one oval.

- Yes *Skip to question 31*
- No *Skip to question 33*

If you are demanding a CT head for this patient,

31. What is the indication according to your opinion? *

____

32. How confident are you to demand head CT in this case? *

Mark only one oval.

	1	2	3	4	5	6	7	8	9	10	
Low	◯	◯	◯	◯	◯	◯	◯	◯	◯	◯	High

Skip to question 34

If you are not demanding head CT in this case,

33. How confident do you feel to not demand head CT in this case? *

Mark only one oval.

	1	2	3	4	5	6	7	8	9	10	
Low	◯	◯	◯	◯	◯	◯	◯	◯	◯	◯	High

CASE - 9

Age	50 years
Triage zone	Red
Mode of injury	Fall (7 metres)
Brief History	Abrasion over frontal region

Time of Incidence	4:00 PM
Time of Presentation	7:00 PM

Airway	Patent, SpO2 - 95% in room air
Breathing	Bilateral equal air entry, RR - 16/m
Circulation	PR - 80/m, BP - 110/60 mm HG, CRT <2 seconds

GCS at presentation	15
GCS at 2 hours of injury	15

Pupils -	Bilateral equal and reactive.
Compression -	Chest – Negative
	Pelvis – Negative
Exposure -	No spine tenderness.
EFAST -	Negative

Any sign of basal skull fracture	-
Post- traumatic seizure	1 episode
Vomiting	-
Loss of consciousness (witnessed)	-
Abnormal drowsiness	-
H/o bleeding or clotting disorders	-
Amnesia	-

34. Is head CT required in this case? *

Mark only one oval.

- ( ) Yes *Skip to question 35*
- ( ) No *Skip to question 37*

If you are demanding a CT head for this patient,

35. What is the indication according to your opinion? *

____

36. How confident are you to demand head CT in this case? *

Mark only one oval.

	1	2	3	4	5	6	7	8	9	10	
Low	⬭	⬭	⬭	⬭	⬭	⬭	⬭	⬭	⬭	⬭	High

Skip to question 38

If you are not demanding head CT in this case,

37. How confident do you feel to not demand head CT in this case? *

Mark only one oval.

	1	2	3	4	5	6	7	8	9	10	
Low	⬭	⬭	⬭	⬭	⬭	⬭	⬭	⬭	⬭	⬭	High

CASE - 10

Age	37 years
Triage zone	Orange
Mode of injury	Hit by rock
Brief History	Injury over occipital region, swelling and crepitation present

Time of Incidence	8:30 PM
Time of Presentation	11:00 PM

Airway	Patent, SpO2 - 90% in room air
Breathing	Bilateral equal air entry, RR - 16/m
Circulation	PR - 88/m, BP - 120/60 mm HG, CRT <2 seconds

GCS at presentation	11
GCS at 2 hours of injury	11

Pupils -	Bilateral equal and reactive.
Compression -	Chest – Negative
	Pelvis – Negative
Exposure -	No spine tenderness.
EFAST -	Negative

Any sign of basal skull fracture	-
Post- traumatic seizure	-
Vomiting	-
Loss of consciousness (witnessed)	-
Abnormal drowsiness	-
H/o bleeding or clotting disorders	-
Amnesia	-

38. Is head CT required in this case? *

Mark only one oval.

- ( ) Yes *Skip to question 39*
- ( ) No *Skip to question 41*

If you are demanding a CT head for this patient,

39. What is the indication according to your opinion? *

____

40. How confident are you to demand head CT in this case? *

Mark only one oval.

	1	2	3	4	5	6	7	8	9	10	
Low	◯	◯	◯	◯	◯	◯	◯	◯	◯	◯	High

Skip to question 42

If you are not demanding head CT in this case,

41. How confident do you feel to not demand head CT in this case? *

Mark only one oval.

	1	2	3	4	5	6	7	8	9	10	
Low	◯	◯	◯	◯	◯	◯	◯	◯	◯	◯	High

CASE - 11

Age	2 months
Triage zone	Red
Mode of injury	Fall (level ground)
Brief History	Injury over left temporal region. No external wounds

Time of Incidence 8:00 PM
Time of Presentation 8:40 PM

Airway	Patent, SpO2 - 98% in room air
Breathing	Bilateral equal air entry, RR - 32/m
Circulation	PR - 78/m, BP - 100/70 mm HG, CRT <2 seconds

GCS at presentation 15
GCS at 2 hours of injury 13

Pupils -	Bilateral equal and reactive.
Compression -	Chest – Negative
	Pelvis – Negative
Exposure -	No spine tenderness.
EFAST -	Negative

Any sign of basal skull fracture	-
Post- traumatic seizure	2 episodes
Vomiting	-
Loss of consciousness (witnessed)	-
Abnormal drowsiness	-
H/o bleeding or clotting disorders	-
Amnesia	-

42. Is head CT required in this case? *

Mark only one oval.

- ( ) Yes *Skip to question 43*
- ( ) No *Skip to question 45*

If you are demanding a CT head for this patient,

43. What is the indication according to your opinion? *

____

44. How confident are you to demand head CT in this case? *

Mark only one oval.

	1	2	3	4	5	6	7	8	9	10	
Low	⬭	⬭	⬭	⬭	⬭	⬭	⬭	⬭	⬭	⬭	High

Skip to question 46

If you are not demanding head CT in this case,

45. How confident do you feel to not demand head CT in this case? *

Mark only one oval.

	1	2	3	4	5	6	7	8	9	10	
Low	⬭	⬭	⬭	⬭	⬭	⬭	⬭	⬭	⬭	⬭	High

CASE - 12

Age	65 years
Triage zone	Red
Mode of injury	Physical assault
Brief History	Multiple bruises over head and body

Time of Incidence 12:00 PM
Time of Presentation 1:00 PM

Airway	Patent, SpO2 - 90% in room air
Breathing	Bilateral equal air entry, RR - 16/m
Circulation	PR - 70/m, BP - 110/60 mm HG, CRT <2 seconds

GCS at presentation 14
GCS at 2 hours of injury 14

Pupils -	Bilateral equal and reactive.
Compression -	Chest – Negative
	Pelvis – Negative
Exposure -	No spine tenderness.
EFAST -	Negative

Any sign of basal skull fracture	-
Post- traumatic seizure	-
Vomiting	-
Loss of consciousness (witnessed)	-
Abnormal drowsiness	-
H/o bleeding or clotting disorders	+
Amnesia	1 hour

46. Is head CT required in this case? *

Mark only one oval.

- Yes *Skip to question 47*
- No *Skip to question 49*

If you are demanding a CT head for this patient,

47. What is the indication according to your opinion? *

____

48. How confident are you to demand head CT in this case? *

Mark only one oval.

	1	2	3	4	5	6	7	8	9	10	
Low											High

If you are not demanding head CT in this case,

49. How confident do you feel to not demand head CT in this case? *

Mark only one oval.

	1	2	3	4	5	6	7	8	9	10	
Low											High

This content is neither created nor endorsed by Google.

Google Forms
