# Supplementary material for: Impact of implementation of the national institute for health and clinical excellence (NICE) head injury guideline in a tertiary care center emergency department: A pre and post-intervention study
Source: PLoS One. 2021 Jul 15;16(7):e0254754. doi: 10.1371/journal.pone.0254754 (PMC8282013; doi:10.1371/journal.pone.0254754)
Supplement: S3 File — (PDF) [file pone.0254754.s003.pdf]

IMPLEMENTING NICE GUIDELINE FOR CT HEAD

PRATISHA PRADHAN
MDGP 1ST YEAR RESIDENT
EMERGENCY DEPARTMENT

Total cases: 18,538

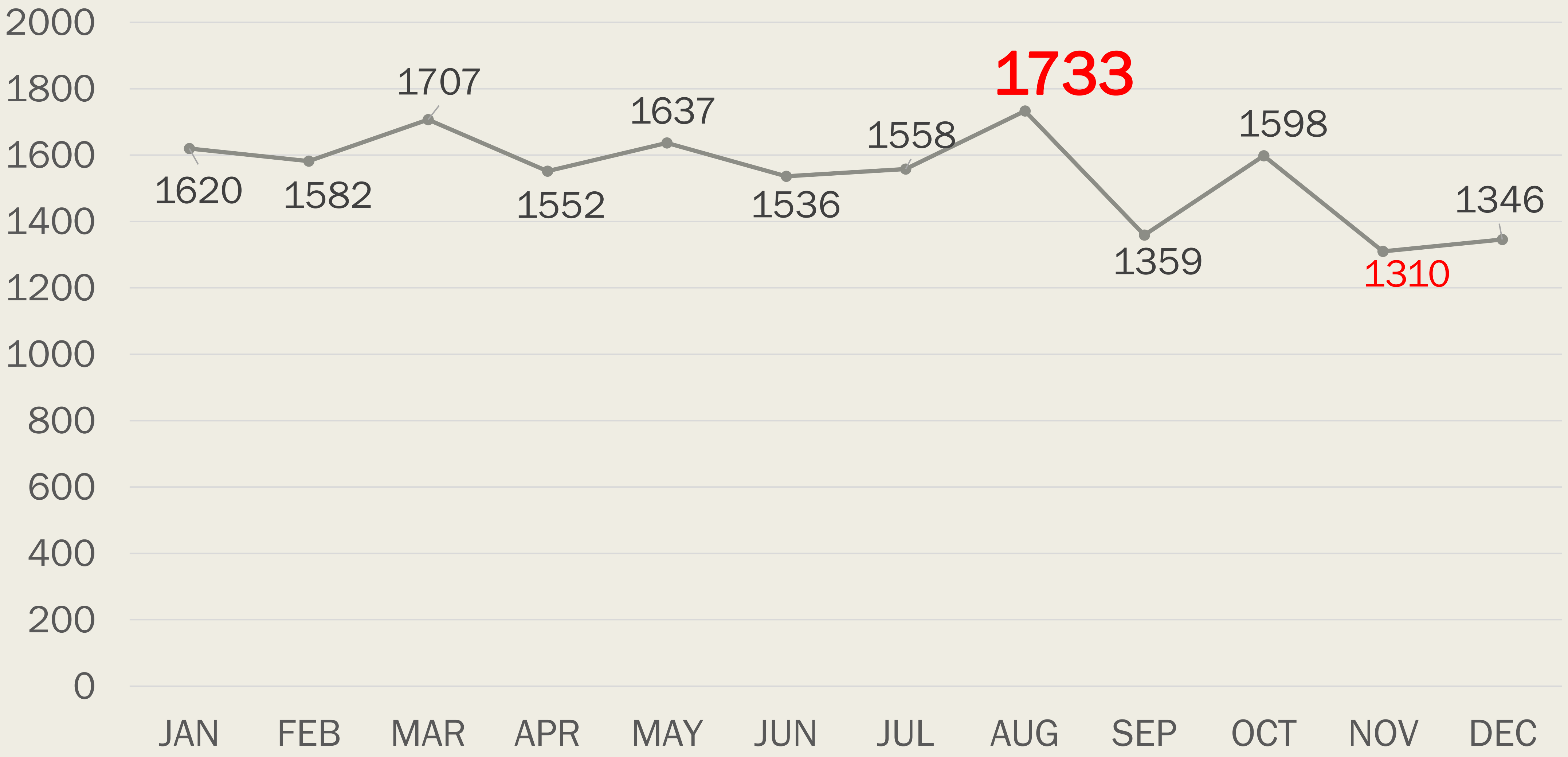

TRAUMA VS NON TRAUMA

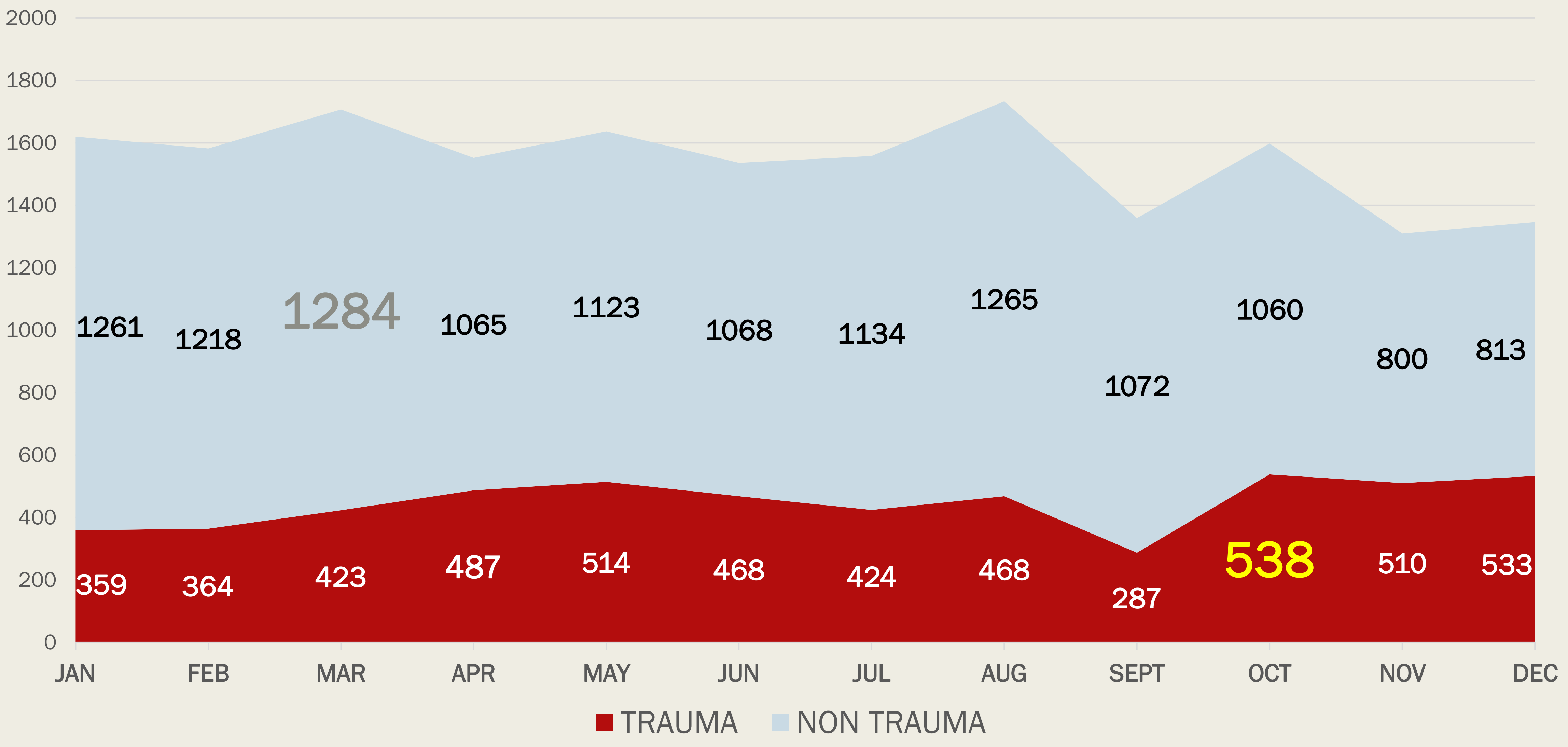

- Formerly known as a **computerized axial tomography scan or CAT scan**
- Computer-processed combinations of many X-ray measurements, taken from different angles,
- Produce cross-sectional (tomographic) images (**virtual "slices**") of specific areas of a scanned object,
- Allowing the user to see inside the object without cutting.
- They use a **narrow X-ray beam** that **circles** around one part of your body.
- A computer uses this information to create a <u>cross-sectional picture</u>.
- This process is <u>repeated</u> to produce a number of slices.

Like one piece in a loaf of bread, this two-dimensional (2D) scan shows a "slice" of the inside of your body.

VIDEO

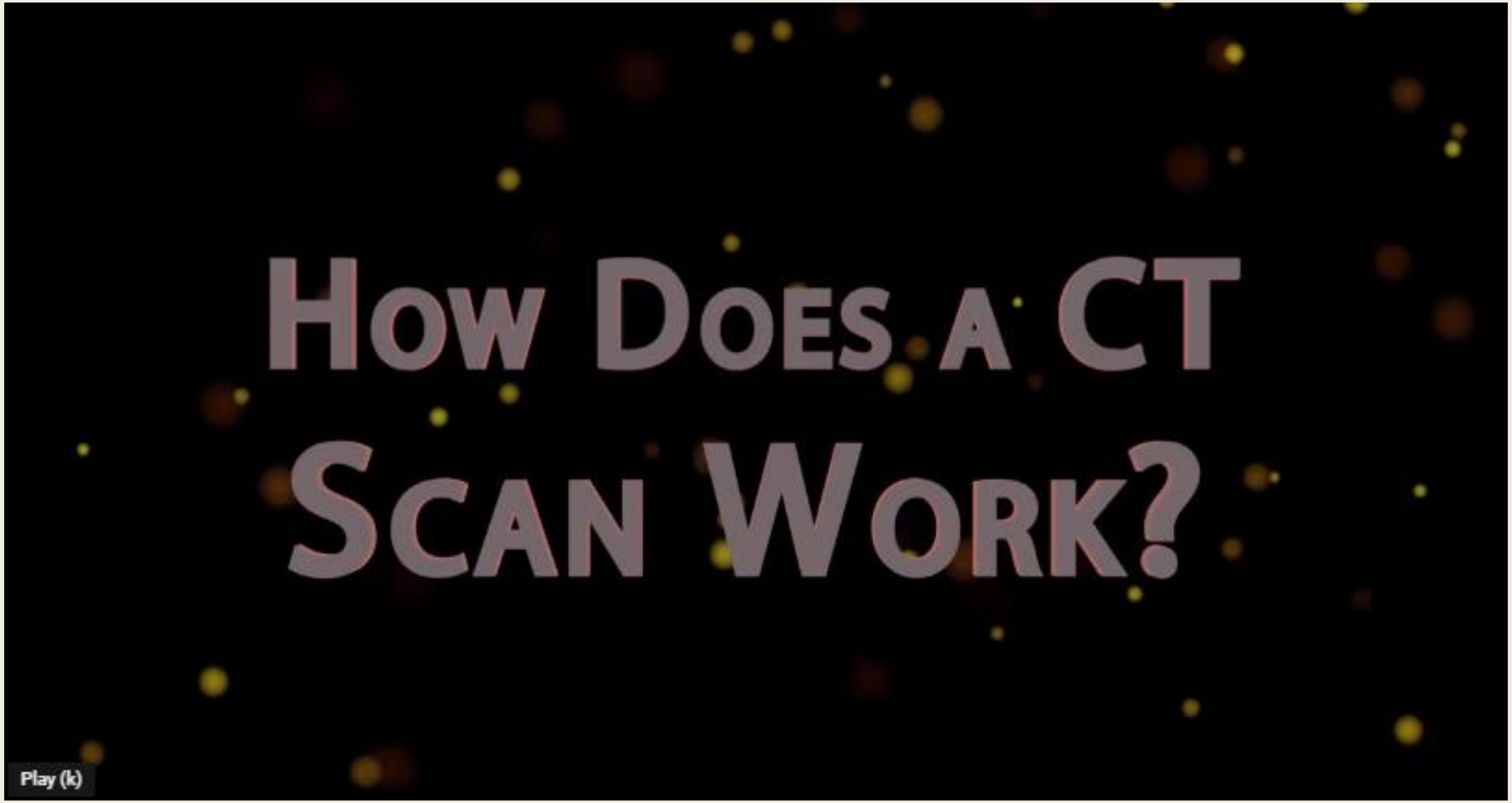

- CT of the head is commonly considered to be the **imaging modality of choice** for the rapid and reliable diagnosis of neurocranial traumatic lesions, such as
  - *Skull fractures,*
  - *Epidural and subdural hematomas, and*
  - *Both hemorrhagic and non- hemorrhagic contusions.*

- Numerous national and international guidelines regarding the use of CT in patients with a minor head injury have been published.

- An important goal of implementing such guidelines is to
  - *Perform CT in only those patients who are at risk of developing complications.*
  - *Reduce costs involved with ct scanning and*
  - *Reduce the strain on emergency, neurology, and radiology departments.*

INTERNATIONAL ATOMIC ENERGY AGENCY

Amount of Radiation Resulting From CT

Examination	Effective Dose (mSv)	Chest X-ray Equivalents
3-view ankle radiography	0.0015	0.07
2-view chest radiography	0.02	1
Radionuclide cystogram	0.18	9
Flouroscopic cystogram	~0.33	~16
Radionuclide bone scan	~5	~250
Brain CT	2	100
Chest CT	up to 3	up to 150
Abdominal CT	up to 5	up to 250

CT scans of the head, expose patients to **~ 1 year** of natural radiation.

This is about **100 times** the radiation dose from a chest X ray.

Radiology

ORIGINAL RESEARCH ■ NEURORADIOLOGY

Minor Head Injury: Guidelines for the Use of CT—A Multicenter Validation Study[1]

Marion Smits, MD
Diederik W. J. Dippel, MD, PhD
Gijs G. de Haan, MD
Helena M. Dekker, MD
Pieter E. Vos, MD, PhD
Digna R. Kool, MD
Paul J. Nederkoorn, MD, PhD
Paul A. M. Hofman, MD, PhD
Albert Twijnstra, MD, PhD
Hervé L. J. Tanghe, MD
M. G. Myriam Hunink, MD, PhD

Purpose: To prospectively and externally validate published national and international guidelines for the indications of computed tomography (CT) in patients with a minor head injury.

Materials and Methods: The study protocol was institutional review board approved. All patients implicitly consented to use of their deidentified data for research purposes. Between February 2002 and August 2004, data were collected in consecutive adult patients with blunt minor head injury (Glasgow Coma Scale score of 13–14 or 15) and a risk factor for neurocranial traumatic complications at presentation at four Dutch university hospitals. Primary outcome was any neurocranial traumatic CT finding. Secondary outcomes were clinically relevant traumatic CT findings and neurosurgical intervention. Sensitivity and specificity of each guideline for all outcomes and the number of patients needed to scan to detect one outcome (ie, the number of patients needed to undergo CT to find one patient with a neurocranial traumatic CT finding, a clinically relevant traumatic CT finding, or a CT finding that required neurosurgical intervention) were estimated.

- The criteria for the use of CT in patients with head injury as set forth by the United Kingdom **National Institute for Clinical Excellence (NICE)** –
- **Highest potential** to reduce the number of CT scans performed while still having reasonable sensitivity for the identification of patients with neurocranial complications or who require neurosurgical intervention after minor head injury.

- For the purposes of the guidelines it was agreed that
  - *Infants are aged under 1 year,*
  - *Children are 1-15 years old and*
  - *Adults are aged 16 years or older.*

NATIONAL INSTITUTE FOR HEALTH AND CARE EXCELLENCE (NICE) 2014 GUIDELINE FOR CT HEAD SCAN

ADULTS

For adults who have sustained a head injury and have any of the following risk factors, perform a CT head scan **within 1 hour of the risk factor** being identified:

- GCS less than 13 on initial assessment in the emergency department.
- GCS less than 15 at 2 hours after the injury on assessment in the emergency department.
- Suspected open or depressed skull fracture.
- Any sign of basal skull fracture (haemotympanum, 'panda' eyes, cerebrospinal fluid leakage from the ear or nose, Battle's sign).
- Post-traumatic seizure.
- Focal neurological deficit.
- More than 1 episode of vomiting.

For adults with <u>any of the following risk factors:</u>

- Age 65 years or older.
- Any history of bleeding or clotting disorders.
- Dangerous mechanism of injury

  A pedestrian or cyclist struck by a motor vehicle,

  An occupant ejected from a motor vehicle,

  A fall from a height of greater than 1 metre or 5 stairs.
- More than 30 minutes' retrograde amnesia of events immediately before the head injury.

Who have experienced **some loss of consciousness or amnesia since the injury**,

Perform a CT head scan **within 8 hours** of the head injury:

CHILDREN

For children who have sustained a head injury and have any of the following risk factors, perform a CT head scan **within 1 hour** of the risk factor being identified:

- Suspicion of non-accidental injury
- Post-traumatic seizure but no history of epilepsy.
- On initial emergency department assessment, GCS less than 14,

  or for children under 1 year GCS (paediatric) less than 15.
- At 2 hours after the injury, GCS less than 15.
- Suspected open or depressed skull fracture or tense fontanelle.
- Any sign of basal skull fracture (haemotympanum, 'panda' eyes, cerebrospinal fluid leakage from the ear or nose, Battle's sign).
- Focal neurological deficit.
- For children under 1 year, presence of bruise, swelling or laceration of more than 5 cm on the head.

For children who have sustained a head injury and have **more than 1 of the following** risk factors (***<u>and none of those in above recommendation</u>***), perform a CT head scan **within 1 hour** of the risk factors being identified:

- Loss of consciousness lasting more than 5 minutes (witnessed).
- Abnormal drowsiness.
- Three or more discrete episodes of vomiting.
- Dangerous mechanism of injury

  High-speed road traffic accident either as pedestrian, cyclist or vehicle occupant,

  Fall from a height of greater than 3 metres,

  High-speed injury from a projectile or other object.
- Amnesia (antegrade or retrograde) lasting more than 5 minutes.

Children who have sustained a head injury and have **only 1 of the risk factors in above recommendation** (and none of those in 1st recommendation)

Should be **observed for a minimum of 4 hours after the head injury.**

If during observation any of the risk factors below are identified, perform a CT head scan **within 1 hour**:

- GCS less than 15.
- Further vomiting.
- A further episode of abnormal drowsiness.

Current warfarin treatment?

If YES,

– *Perform CT head scan within 8 hours of head injury.*

A provisional written radiology report should be made available within 1 hour of the scan being performed.

If none of these risk factors occur during observation,

use clinical judgement

to determine whether a longer period of observation

is needed.

Can I undergo a CT scan while I am pregnant?

- **Yes, if medically justified and with certain precautions.**
- The aim is to minimize the unborn child's radiation exposure.
- An unborn child is considered to be more sensitive than adults or children to potential adverse radiation effects.
- In many examinations such as CT of the head (including dental CT scans), chest and limbs, the pelvic region is not in the direct beam and the <u>dose to the unborn child can be very low.</u>
- If the procedure is essential to the mother's health, the doctors take special actions to keep the dose to the unborn child as low as possible.

EXAMPLE

For **children** who have sustained a head injury and have any of the following risk factors:

Suspicion of non-accidental injury	
Post-traumatic seizure but no history of epilepsy.	
On initial emergency department assessment,<br>Age 1 year to 15 years: GCS < 14<br>Children < 1 year: GCS (pediatric) < 15.	
At 2 hours after the injury, GCS less than 15.	
Suspected open or depressed skull fracture or tense fontanelle.	
Any sign of basal skull fracture (haemotympanum, 'panda' eyes, and cerebrospinal fluid leakage from the ear or nose, Battle's sign).	
Focal neurological deficit.	
For children under 1 year, presence of bruise, swelling or laceration of more than 5 cm on the head.	

Children who have sustained a head injury and have only 1 of the risk factors stated above, observe for a minimum of 4 hours after the head injury. If during observation any of the risk factors below are identified:

GCS less than 15.	
Further vomiting.	
A further episode of abnormal drowsiness.	

THANK YOU!
