# Supplementary material for: Impact of implementation of the national institute for health and clinical excellence (NICE) head injury guideline in a tertiary care center emergency department: A pre and post-intervention study
Source: PLoS One. 2021 Jul 15;16(7):e0254754. doi: 10.1371/journal.pone.0254754 (PMC8282013; doi:10.1371/journal.pone.0254754)
Supplement: S4 File — (PDF) [file pone.0254754.s004.pdf]

NICE National Institute for Health and Care Excellence

Algorithm 1: Selection of adults for CT head scan

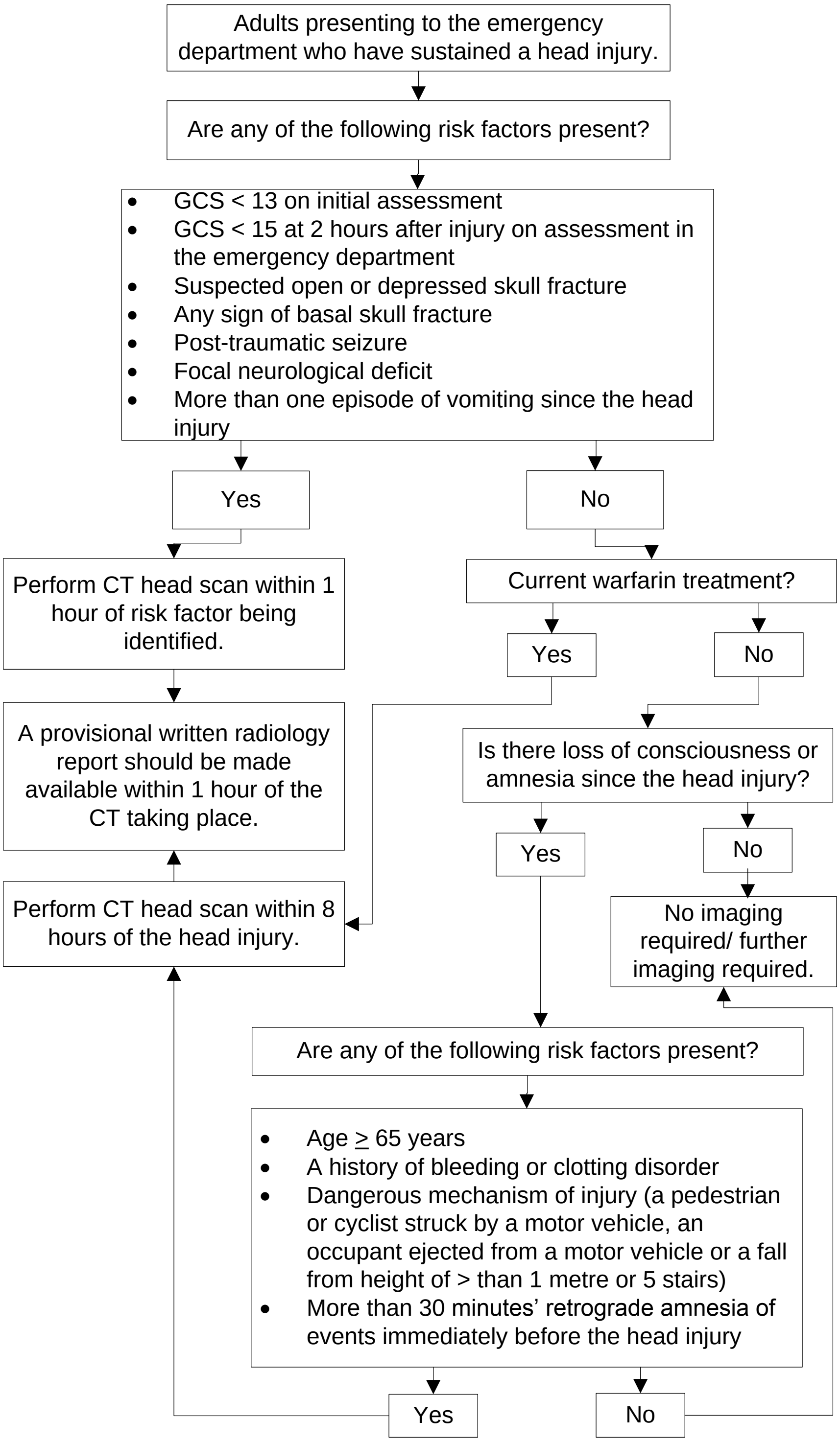

© National Institute for Health and Care Excellence, 2014. 'Head injury', NICE clinical guideline 176. London: National Clinical Guideline Centre (www.guidance.nice.org.uk/CG176)

Algorithm 2: Selection of children for CT head scan

Children presenting to the emergency department who have sustained a head injury.

Are any of the following risk factors present?

- Suspicion of non-accidental injury
- Post-traumatic seizure, but no history of epilepsy
- On initial assessment GCS <14, or for children under 1 year GCS (paediatric) < 15
- At 2 hours after the injury GCS < 15
- Suspected open or depressed skull injury or tense fontanelle.
- Any sign of basal skull fracture (haemotympanum 'panda' eyes, cerebrospinal fluid leakage from the ear or nose, Battle's sign).
- Focal neurological deficit
- For children under 1 year, presence of bruise, swelling or laceration of more than 5 cm on the head

Yes

Perform CT head scan within 1 hour of risk factor being identified. A provisional written radiology report should be made available within 1 hour of the CT head scan taking place.

No

Are any of the following risk factors present?

- Witnessed loss of consciousness > 5 minutes
- Abnormal drowsiness
- 3 or more discrete episodes of vomiting
- Dangerous mechanism of injury (high-speed road traffic accident either as a pedestrian, cyclist or vehicle occupant, fall from height of > 3 metres, high speed injury from an object
- Amnesia (antegrade or retrograde) lasting > 5 minutes (assessment not possible in pre-verbal children and unlikely in any child < 5 years).

Yes, > 1 factor

Yes, 1 factor

No

Observe for a minimum of 4 hours post head injury.

Are any of the following risk factors present during observation?

- GCS < 15,
- further vomiting
- further episodes of abnormal drowsiness

Yes

No

Current warfarin treatment?

Yes

No

Perform CT head scan within 8 hours of the injury. A provisional written radiologist's report should be made available within 1 hour of the CT head scan taking place.

No imaging required. Use clinical judgement to determine when further observation is required.

© National Institute for Health and Care Excellence, 2014. 'Head injury', NICE clinical guideline 176. London: National Clinical Guideline Centre (www.guidance.nice.org.uk/CG176)

NICE National Institute for Health and Care Excellence

Algorithm 3: Selection of adults for imaging of the cervical spine

Adults presenting to the emergency department who have sustained a head injury.

Are any of the following risk factors present?

- GCS < 13 on initial assessment
- Intubation
- A definitive diagnosis of cervical spine injury is required urgently (e.g. before surgery)
- Other body areas are being scanned for head injury or multi-region trauma
- The patient is alert and stable, there is a suspicion of cervical spine injury and any of the following are present: age > 65; dangerous mechanism of injury (fall from > 1 metre or 5 stairs, axial load to head [e.g. diving], high speed motor vehicle collision, rollover motor accident, ejection from a motor vehicle, accident involving motorized recreational vehicles, bicycle collision); focal peripheral neurological deficit or paraethesia in the upper or lower limbs.

Yes

Perform CT cervical spine within 1 hour of risk factor being identified.

A provisional written radiology report should be made available within 1 hour of the CT taking place.

No

Is there suspicion of cervical spine injury (neck pain or tenderness) with any of the following low risk factors present, which indicate it is safe to assess the range of movement of the neck?

- Involved in a simple rear–end motor vehicle collision
- Is comfortable in a sitting position in the emergency department
- Has been ambulatory at any time since injury
- Absence of midline cervical tenderness
- Presents with delayed onset of neck pain

No

Perform three-view cervical spine X-rays within 1 hour of risk factor being identified.

Are the three-view cervical spine X-rays technically inadequate, suspicious or definitely abnormal?

Yes

No

Yes

On assessment can the patient actively rotate the neck to 45 degrees to the left and right?

No

Yes

No imaging/further imaging required

© National Institute for Health and Care Excellence, 2014. 'Head injury', NICE clinical guideline 176. London: National Clinical Guideline Centre (www.guidance.nice.org.uk/CG176)

NICE National Institute for Health and Care Excellence

Algorithm 4: Selection of children for imaging of the cervical spine

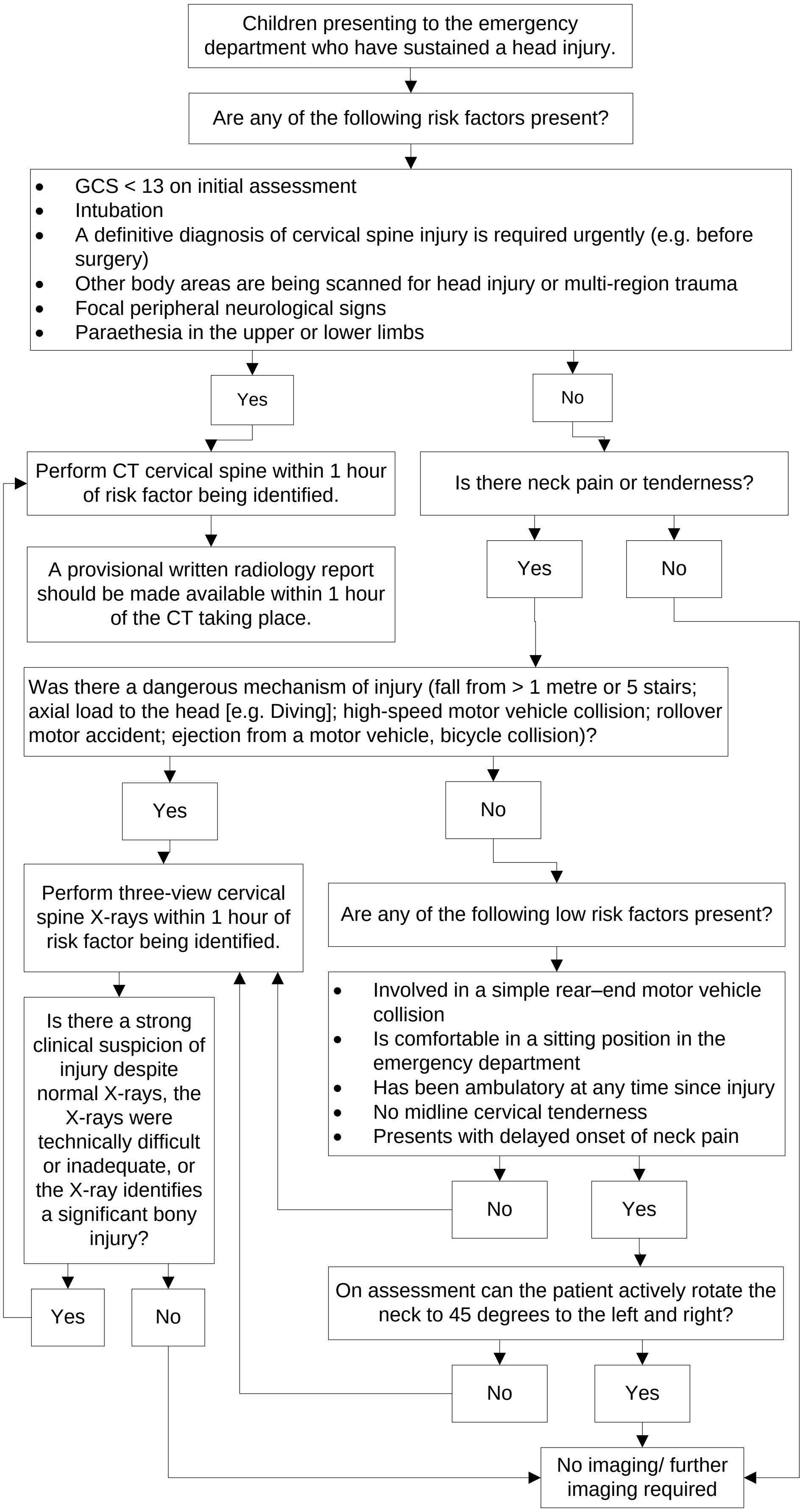

© National Institute for Health and Care Excellence, 2014. 'Head injury', NICE clinical guideline 176. London: National Clinical Guideline Centre (www.guidance.nice.org.uk/CG176)
